# Supplementary material for: Data sharing practices in collaborative human genomic research in low- and middle-income countries: A systematic review protocol
Source: PLoS One. 2023 Nov 2;18(11):e0292996. doi: 10.1371/journal.pone.0292996 (PMC10621801; doi:10.1371/journal.pone.0292996)
Supplement: Attachment — Submitted filename: Revised point by point.docx [file pone.0292996.s002.docx]

**Data sharing practices in** **collaborative human genomic research in low- and middle-income countries: A systematic review protocol**

**Editor:** Please ensure that your manuscript meets PLOS ONE's style requirements,

**Author:** Thank you for observing this. The manuscript has been revised to ensure it meets PLOS ONE’S style requirements

**Editor:** We noticed you have some minor occurrence of overlapping text with the following previous publication(s), which needs to be addressed: In your revision ensure you cite all your sources (including your own works), and quote or rephrase any duplicated text outside the methods section. Further consideration is dependent on these concerns being addressed.

**Author:** While there is overlap, the study will address some of the recommendations made and all the studies have Yes, been referenced Lines (110-114)

Editor: We note that the grant information you provided in the ‘Funding Information’ and ‘Financial Disclosure’ sections do not match. When you resubmit, please ensure that you provide the correct grant numbers for the awards you received for your study in the ‘Funding Information’ section

Author: Thank you for catching this. I will ensure the correct grant number is provided both for the funding and financial disclosure

Reviwer 1: Ekusai-Sebatta et al, designed a systematic review protocol to generate information which will highlight the context of data sharing in LMIC to policy makers and funders in Sub-Saharan Africa. The authors aim to investigate data sharing perceptions and practices amongst researchers, participants, and regulators in LMIC who are involved in human genomic collaborative research. These authors aim to conduct an electronic search of existing databases and repositories for published and grey literature focused on data sharing, using the recommended PRISMA-P checklist.This is original work and has not been started yet. The authors have contextualised their proposed protocol along PRISMA-P guidelines. They have referenced relevant published work on the subject

Author: Thank you so much. The authors appreciate this positive feedback

Major issues

Reviewer 1: The authors indicate that they have registered this protocol in PROSPERO and will share the full report with relevant stakeholders to ensure a wide reach. However, the authors do not indicate HOW they will disseminate the full report to accomplish this – I feel that this is important to specify in the protocol. PRISMA refers to the Open Science Framework

**Author:** Thank you. We have included both in the abstract and the methods section that PRISMA refers to the Open Science Framework (lines 31, 135-136).

**Author:** The authors also state that Data sharing is not applicable at this point of the study, however, it is a keyword for this protocol, and it is recommended that the authors indicate where/when the data generated by the electronic searches will be made accessible.
According to PLOS guidelines: In the Data Availability Statement, briefly describe how data generated will be made accessible when the study is completed.

**Author:** The authors apologize for making such a grave error. In the data availability report, a statement has been included indicating that the systematic review was registered in PROSPERO and the registry number was included in their abstract (CRD42022297984) line

32.

Also, the authors have indicated under data sharing that “The search strategy is attached as an additional file. Additional data sources will be available on request. Requests can be made to the corresponding author at: [ekusai@gmail.com](mailto:ekusai@gmail.com)” (Lines 341-343).

**Reviewer 1:** Risk of Bias: ln 283 – is it the abstraction or extraction process?

Author: Thank you. This has been changed to the data extraction process lines 229

**Reviewer 1:** ln 306- Ethics approval and Consent to participate – This subheading seems inappropriate for the text following it.

**Author:** Information has been included under the subheading Consent to participate and it now reads “There is no individual respondent data in the manuscript”. Lines 321

**Reviewer1:** Reporting and Dissemination
Ln 285-287 – This paragraph is repeated almost verbatim in the Discussion Ln 302-305 and should preferably be reworded.

Author:Thank you for catching this. The repeated sentence has been deleted from the discussion

**Other issues**

In the abstract, EMBASE and MEDLINE are not mentioned as data sources. Is there a reason for this?

**Author:** Thank you. EMBASE and MEDLINE have been included in the abstract. (Lines 33-34)

**Reviewer 1:** Table 1: Should the word funders be included in Description of Population

**Author:** The word funders has been added to population (Table 1)

**Reviewer 1:** Authors should consider adding intermediate data to the Description of intervention/ exposure

**Author:** Thank you. Intermediate data has been added to the exposure (Table 1)

**Reviewer 1:** Minimizing bias.
Ln 222- please clarify (i) whether this is extraction or abstraction.(ii) please indicate whether it is the authorship team who will resolve differences by discussion and consensus

**Author:** Thank you. It is during abstraction. Yes, the authorship team will provide discussion and consensus. (Line 227).

**Reviewer 1:** Ln 224- The subheading “Data abstraction and coding’’ should rather read ‘Data extraction and coding’’ because the paragraph discusses extraction.

**Author:** Thank you.It is data is data extraction.

**Reviewer 1:** Ln 229- Indicate who the senior reviewer is (EM)

**Author:** The senior reviewer is EM (Line 234)

**Reviewer:** The authors should consider listing the ten components that will be assessed for methodological rigour.
Ln 234- This should read “No studies WILL BE eliminated…..” as this work is still to be done

**Author:** The nine components of the Hawker checklist have been listed. (lines 238-243). The nine components include: 1) Title and abstract, 2) introduction and aims, 3) method and data, 4) sampling, 5) data analysis, 6) ethics and bias, 7) results, 8) transferability, 9) implications and usefulness

**Reviewer 1:** Data synthesis Ln 254 – please reference ‘’a code book’’

**Author:** A code book has been referenced (Line 261).

**Reviewer 1:** Ln 264 – please indicate who will guide discussions to resolve the disagreement? My feeling is to avoid a negative word such as ‘disagreement’ – instead, you could say that ‘’differences of opinion will be resolved in guided team discussions’’. Indicate who will guide these sessions.

**Author:** Thank you. This has been changed and now reads “Differences in opinion will be resolved through discussions guided with the team. (Lines 271-272).

Other minor issues

**Reviewer 1:**

Ln 52 – please clarify what is meant by ‘’óther’’ investigators

**Author:** Thank you. I have edited it and it now reads “Data sharing is the practice of making research data available to other investigators conducting similar work”.(Lines 53-54).

**Reviewer 1**: Ln 69- please indicate that the protocol is for data sharing of human genomic data

**Author:** Thank you. This has been indicated (Line 77)

Reviewer 1: Ln 82- SSA never appears in full before this line.

**Author**: This was an error. The word SSA has been removed (Line 83)

**Reviewer 1**: Ln 111- ‘’with these policies……’’ does not follow from the previous sentence

**Author**: This has been moved up to Lines immediately following previous sentence on policies (Lines 104-107)

Reviewer 1: Ln 121 and 123- be consistent throughout with the term genomic or genomics research

**Author**: Thank you. This has been corrected

**Reviewer 2:**

I believe the systematic review of data sharing practices are indeed warranted in the era of genomics research. I am concerned however how much data the authors will be able to identify given that often details related to MTA or data sharing are not included in papers. HOWEVER, often this is implicit in the ethics obtained from specific institutions. Given that this information is not always included in papers how would the authors reconcile the data noted in publications to their objectives? I therefore suggest it is also important to understand the governance structures and legislation of the institutions and countries included in the papers to gain an understanding of potential practices of researchers in genomics. The assumption the authors are making includes that data is only going in one direction and it would be important to note the direction of data sharing between continents and within continents and how this differs. My main recommendation is that authors include strategies to overcome some of the important missing data related to practice of data sharing and sharing of biological samples.

**Author:** Thank you so much. You raise very important issues. While some of the information about data sharing maybe indeed be found in the methods section, not all researchers may write about it. The authors will look at the governance structures of the different institutions.

Yes, indeed it is an important observation because since data sharing takes places between continents, knowledge of the direction is important. Also, through the review, we would like to find out how data data sharing is happening between countries in the global south. A statement on this recommendation has been made in the protocol and now reads “An understanding of the direction data sharing takes between continents, within countries within the global south and how this differs is crucial” (Lines 101-102). Some of the strategies to overcome data sharing include contacting authors to reconcile some of the missing data and checking institutional websites.
